# Virulence determinants of *Fusarium oxysporum* for clinical infections

**DOI:** 10.1371/journal.ppat.1014630

**Published:** 2026-10-01

**Authors:** Jeffrey J. Coleman, Neta Shlezinger, Li-Jun Ma

**Affiliations:** 1 Department of Entomology and Plant Pathology, Auburn University, Auburn, Alabama, United States of America; 2 Koret School of Veterinary Medicine, The Hebrew University of Jerusalem, Rehovot, Israel; 3 Department of Biochemistry and Molecular Biology, University of Massachusetts-Amherst, Amherst, Massachusetts, United States of America; Vallabhbhai Patel Chest Institute, INDIA

## Clinical relevance of *Fusarium*

Fungi within the genus *Fusarium* are known plant pathogens that cause devastating diseases on many agriculturally important crops [1,2]. However, they are also important animal and human pathogens and are recognized as one of the high-priority fungal infectious agents responsible for invasive fungal diseases by the World Health Organization [3]. While this diverse genus is currently divided into 23 species complexes [4], only a few of these are associated with human infections. Most notably, the *F. solani* species complex (FSSC) and *F. oxysporum* species complex (FOSC) have been estimated to account for ~80% of all clinical *Fusarium* infections [5]. Within these species complexes, genotypes of *Fusarium* associated with clinical infections can be genetically diverse. For instance, at least 26 sequence types (STs) of *F. oxysporum* were identified among clinical infections with different frequencies [6]. Among clinical isolates, ST 33 is the most common worldwide, and is frequently associated with plumbing systems [7], including hospital waters [8].

*Fusarium* is one of the most common etiological agents responsible for fungal keratitis [9]. Able to form a complex biofilm on contact lenses facilitating disease development [10], *Fusarium* was responsible for a multistate contact lens-associated keratitis outbreak [11,12]. *Fusarium* infections in immunocompetent individuals are usually superficial, causing localized infections such as nail infections termed onychomycosis. However, cases of invasive or disseminated infections are increasing in frequency with the growing immunocompromised patient population [13]. Patients with hematological malignancies, neutropenia, or those who have undergone transplantation, in particular hematopoietic stem cell transplant patients, have an increased risk of developing severe life-threatening invasive infections or disseminated fusariosis [13,14]. It is postulated that most invasive *Fusarium* infections begin through inhalation of the conidia into the pulmonary system, which then germinate and colonize the alveolar tissue [13]. Alternatively, disruption of the integrity of the skin by physical trauma, ulcers, burns, or catheter-associated open wounds, provide an opportunity for *Fusarium* to infect [13]. Infections by the fungus may present in a wide range of symptoms for a patient, where some of the variables include patient immune status, location of infection, and likely the specific fungal isolate responsible for the infection. Many of these variables have been recently discussed by Nucci and Anissie, 2023, and we refer the reader to this review article for specific information regarding epidemiology and clinical presentation of *Fusarium* infections.

The intrinsic resistance to many commonly prescribed antifungal agents, including the echinocandins and some of the triazoles, contributes to the high rate of mortality associated with fusariosis within specific patient populations [13,15]. As observed in other medically relevant fungi, multiple factors are involved in increased antifungal resistance in *Fusarium.* The genomes of *Fusarium* carry three copies of the *CYP51* gene [16], also widely known as *ERG11*, that encodes the cytochrome P450 sterol 14α-demethylase. A key enzyme in fungal ergosterol biosynthesis and the target of azole drugs, the increased copy number of *CYP51* likely contributes to increased resistance to these therapeutic drugs. Expression studies after treatment with antifungal agents have shown up-regulation of genes involved in ergosterol biosynthesis and membrane biosynthetic pathways, as well as multiple efflux pumps [17]. An ABC transporter involved in cellular removal of antifungal agents and an NADPH-cytochrome P450 reductase that supplies the necessary electrons for ergosterol biosynthesis have been directly shown to increase antifungal resistance [18,19].

Despite the medical importance recognized by the WHO, *Fusarium* is understudied as a clinically important fungal pathogen. Only recently have genomes become publicly available for *Fusarium* isolates of clinical origin [16,20], and subsequent functional genomic studies are just beginning to be implemented [21,22]. Challenges that have delayed the exploration of *Fusarium* as a medically relevant pathogen include: (1) the vast genetic diversity among clinical isolates; (2) limitations in the number of dominant selectable markers available for genetic screens; (3) the intrinsic resistance of the fungi to many compounds; and (4) our limited understanding of the cross-kingdom nature of this group of fungal pathogens that includes many agriculturally important plant pathogens.

While research into the clinical aspect of these fungi is still in its infancy, evidence is rapidly accumulating to support the existence of genetic bases underlying human infection. First, even though the human-infecting FOSC strains are quite diverse (26 STs), a single dominant lineage is responsible for a majority of reported clinical cases, ST 33 and closely related STs such as ST 128, which is evident in the limited epidemiological studies of global reported outbreaks [8,12,23]. This implies that some genetic factor(s) associated with this lineage may contribute to infection. In addition, potential virulence factors for mammalian infections have been identified in some *Fusarium* species and isolates [16,21].

## The *Fusarium* accessory genome, adaptability, and virulence

The notion that there are genetic determinants that contribute to FOSC virulence to humans is also supported by their genomic information. One distinct genomic feature recognized in both FSSC and FOSC genomes is genomic compartmentalization. A genome within these species complexes can be partitioned into two categories: the core genome, encompassing genes conserved within the species complex involved in essential cellular processes, and the accessory genome harboring nonessential genes that are rapidly evolving.

In plant pathogenic isolates of *F. oxysporum*, it is within the accessory genome that host-specific virulence factors are enriched and therefore influence the host range of the isolate. The accessory genome can be organized into entire chromosomes, referred to as accessory chromosomes, or genomic regions usually located in the sub-telomeric regions of core chromosomes [24,25]. Importantly, accessory chromosomes have experimentally been demonstrated to be laterally transferred from one isolate to another, thus transferring the virulence [24]. Conversely, accessory chromosomes of an individual isolate can be spontaneously lost, and therefore the isolate can lose its host-specific virulence [26].

As with plant pathogenic isolates, clinical isolates within the FOSC also carry distinct sets of accessory chromosomes encoding putative virulence factors against mammalian hosts [16]. Four unique accessory chromosomes not present in plant pathogenic FOSC isolates were reported in the genome of an FOSC isolate from a fusariosis patient (Fig 1). The 5.3 Mb accessory genome of this isolate encoded 812 putative genes and was enriched for genes involved in metal ion and cation transport and responses to extracellular stimuli. Specifically, these genes are significantly enriched for metal ion binding and oxidation-reduction, including a homolog of human ceruloplasmin (CP), a major copper-carrying protein in the blood that is essential for normal iron homeostasis [16], which may function in circumventing host nutritional immunity. Of particular interest was an increase in the number of *PacC* homologs in the accessory genome. PacC is an important transcription factor that is involved in controlling the fungal response to changes in pH and *ΔpacC* mutants are significantly reduced in virulence in a murine model of infection [27]. The increased copy number may offer an adaptive advantage for the pathogen under alkaline mammalian host pH conditions. Interestingly, the paralogous copies of *PacC* in the accessory genome were flanked by transposable elements [16]. Experimental evolution studies of FOSC isolates have documented that stressful conditions induce transposable element activity and their dynamic nature can result in increased fungal fitness [28]. Not present in plant pathogenic strains, the accessory genome of this fusariosis strain is partially conserved in another clinical FOSC isolate from a keratitis patient [16]. The ~140 kb shared region, which includes one of the *PacC* paralogs, suggests there could exist common loci within the accessory genome that play a role in pathogenesis on mammalian hosts.

**Fig 1:**
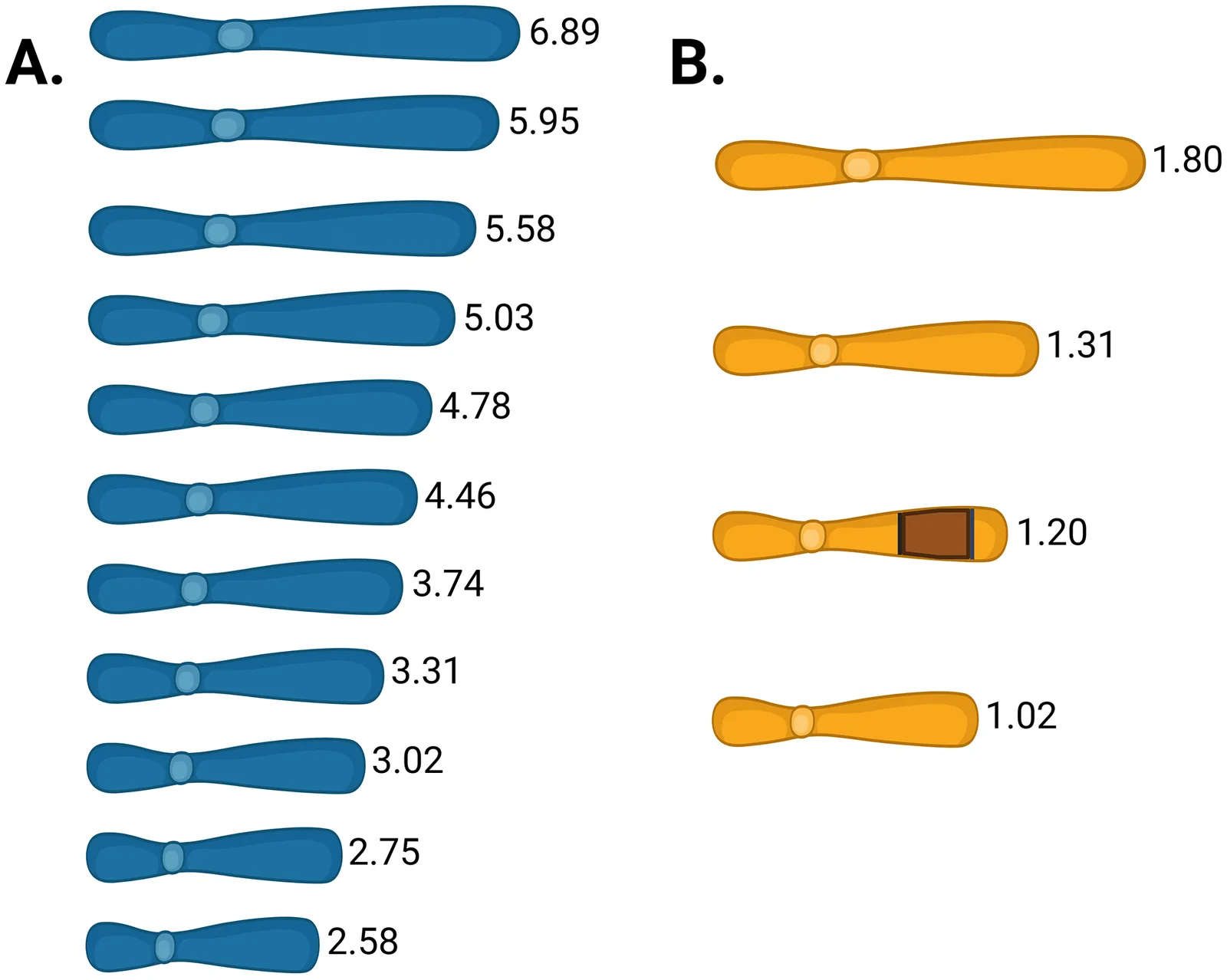
Schematic representation of the genome organization of clinical isolate ST 128 NRRL 32931. **(A)** the eleven core chromosomes and their sizes (Mb) and **(B)** the four accessory chromosomes and their sizes (Mb). The dark area on the 1.20 Mb accessory chromosome is shared with the accessory genome of another sequenced clinical isolate MRL8996 (NRRL 47514) and encodes a *PacC* homolog. Figure created in BioRender.

The genotypic distinctions carried by accessory genes between the plant pathogenic and human pathogenic isolates are also reflected in phenotypic assays. Compared to strains isolated from plant hosts, clinical strains displayed increased thermotolerance, reflecting the adaptation of these human pathogens to the mammalian environment [21]. An in vivo assay using a mouse keratitis model demonstrated the ability to cause severe corneal colonization by the keratitis strain [21]. Both the clinical *F. oxysporum* fusariosis strain and the keratitis strain exhibited enhanced virulence when compared to a plant pathogenic strain in an immunocompromised mouse model, as measured by animal survival kinetics, tissue damage, and cytotoxicity to mammalian host cells associated with inflammatory dysregulation and impaired cellular recruitment [22].

The evidence of increased thermotolerance and virulence on a mammalian host in conjunction with a conserved core genome and distinct accessory chromosome, supports the role of the accessory genome in pathogenicity on mammals.

## Virulence factors of *F. oxysporum* in clinical infections

While a plethora of general and host-specific virulence factors have been reported in isolates of the FOSC with respect to plant pathogenicity, only a few have been confirmed to be involved in pathogenesis in a murine model of infection. A complex trait controlled by diverse biological processes, most of the known genes conferring virulence to mammalian hosts can be classified into three groups: (1) regulation of cellular signaling (i.e., transcription factors/regulators), (2) secreted effectors and secondary metabolites, and (3) maintaining heavy metal homeostasis (Table 1). Currently, most reported virulence factors with experimental validation are encoded in the core genome. A highly promising research area, the contribution of individual genes from the accessory genome of clinical FOSC isolates remains largely unexplored.

**Table 1:** Summary of confirmed virulence factors contributing to disease development in *F. oxysporum.*

Virulence factor	Function	Reference(s)
**Cellular Signaling**		
PacC	Transcription factor regulating pH response	[27]
Fmk1	MAP kinase	[27,30]
Fgb1	G-protein β subunit	[30]
WC-1	Photoreceptor involved in light signal transduction	[31]
VeA, VelB, VelC, LaeA	Members of the velvet regulatory complex	[32]
**Secreted proteins and secondary metabolites**		
Fpr1	Secreted protein in CAP superfamily – hypothesized to be involved in tissue invasion and/or evading host immune system	[29]
Fub1	The key polyketide synthase responsible for fusaric acid biosynthesis	[33]
SidC, SidD	Each is a key nonribosomal peptide sythetase responsible for production of the siderophores ferricrocin and triacetylfusarinine, respectively	[32]
**Heavy metal homeostasis**		
HapX	A bZIP transcription factor	[35,36]
CrpF	Copper transporter	[37]
Mt1	Metallothionein	[38]

One of the most noteworthy virulence factors reported in *F. oxysporum* is the pathogenesis-related protein Fpr1 [29]. This secreted protein belongs to the CAP protein superfamily (PF00188), including mammalian Cysteine-rich secretory proteins, insect Ag5-antigens, and plant PR-1 proteins. This effector is found in many ascomycete fungi and is remarkably expanded in *Candida albicans* with five members. The expression of this effector is induced when cultivating *F. oxysporum* in human blood and in an in vivo mouse model. Even though the mechanism remains unknown, Fpr1 is required for full virulence and it is speculated to be involved in promoting tissue invasion and/or evading the host immune system [29].

The induction of the effector Fpr1 is under the control of the MAP kinase Fmk1 [30]. While mutants of *fmk1* maintain wild-type levels of virulence [27], a double mutant with the G-protein β subunit *fgb1* is significantly reduced in virulence, indicating multiple signaling pathways collectively coordinate mammalian virulence [30].

In addition to the PacC transcription factor described above, other reported regulators involved in *F. oxysporum* pathogenicity include the photoreceptor white collar 1 (WC-1). Even though WC-1 functions in perceiving light and subsequently controlling aerial hyphae, hydrophobicity, and carotene production, a *wc-1* mutant is reduced in virulence when compared to the parental wild-type [31], which is hypothesized to be caused by an increased production of secondary metabolites.

Another critical regulator involved in pathogenicity is the velvet complex, composed of VeA, VelB, VelC, and LaeA. VeA and LaeA are required for full virulence of *F. oxysporum* on both tomato plants and in an immunosuppressed mouse model [32]. This regulatory complex is involved in many developmental aspects of the fungus, including growth, conidiation, as well as chromatin architecture and secondary metabolite production. Biosynthesis of fusaric acid is abolished in the *ΔveA* and *ΔlaeA* mutants [32]. Mutation of *FUB1*, the gene encoding the essential polyketide synthase responsible for biosynthesis of fusaric acid, also results in a reduction in virulence [33], although to a lesser degree than mutation of the velvet complex subunits suggesting other factors contributing to virulence are under transcriptional control of the velvet complex.

One of the other potential factors under control of the velvet complex that could contribute to virulence is the production of the siderophores ferricrocin and triacetylfusarinine C, which are synthesized by the nonribosomal peptide sythetases SidC and SidD [32]. Production of siderophores and their role in maintaining iron levels have been well established as important virulence factors for a number of medically important fungi [34]. In support of the importance of iron acquisition during *F. oxysporum* infection, deletion of the bZIP transcription factor HapX, which regulates iron homeostasis, results in an avirulent mutant that is limited in its ability to colonize the kidneys and lungs of infected animals [35]. Additionally, a *ΔhapX* strain exhibits significantly decreased corneal opacification and growth when compared to the WT isolate in an interstromal mouse model of infection [36]. Proteins involved in copper acquisition have also been shown to be involved in virulence on mammals, as mutants of the copper transporter CrpF are increased in virulence [37], and mutants of the metallothionein Mt1 display reduced survival in macrophages although there is no significant difference in virulence [38].

More recently, an additional potential mechanism linking fungal metabolism to virulence has been proposed. A recent preprint reported a role for sterol metabolism in shaping virulence of *F. oxysporum* in mammalian hosts [22]. Clinically derived isolates were found to exhibit altered ergosterol homeostasis, including increased extracellular ergosterol release, which was associated with enhanced pathogenicity in a murine infection model. Mechanistically, extracellular ergosterol promoted macrophage cytotoxicity through caspase-1-dependent pyroptosis and modulated inflammatory signaling by inducing pro-inflammatory cytokines while suppressing chemokine production, resulting in impaired neutrophil recruitment. These findings suggest that fungal sterol metabolism may contribute to virulence not only through its role in membrane biology but also by directly influencing host immune responses.

## Models to study *Fusarium* infection

There is a growing interest in studying *Fusarium* spp. as medically important fungi and models to evaluate and study their virulence are rapidly developing (Fig 2). Multiple mouse models of infection have been established for use with members of the FOSC. Systemic models of fusariosis based on intravenous infection of neutropenic mice have been used to study disseminated disease, in which chlamydospores, the survival spore of *Fusarium*, are produced in infected tissues [39]. Murine models of pulmonary infection have also been developed using intranasal or intratracheal inoculation, resulting in rapid disease progression and high mortality that underscore the capacity of *Fusarium* to cause severe respiratory disease in immunocompromised hosts [40]. A contact lens-associated model of infection for *Fusarium* keratitis has been established and demonstrated that hyphae preincubated on the contact lens could establish a corneal infection within 24 hours [21,41].

**Fig 2:**
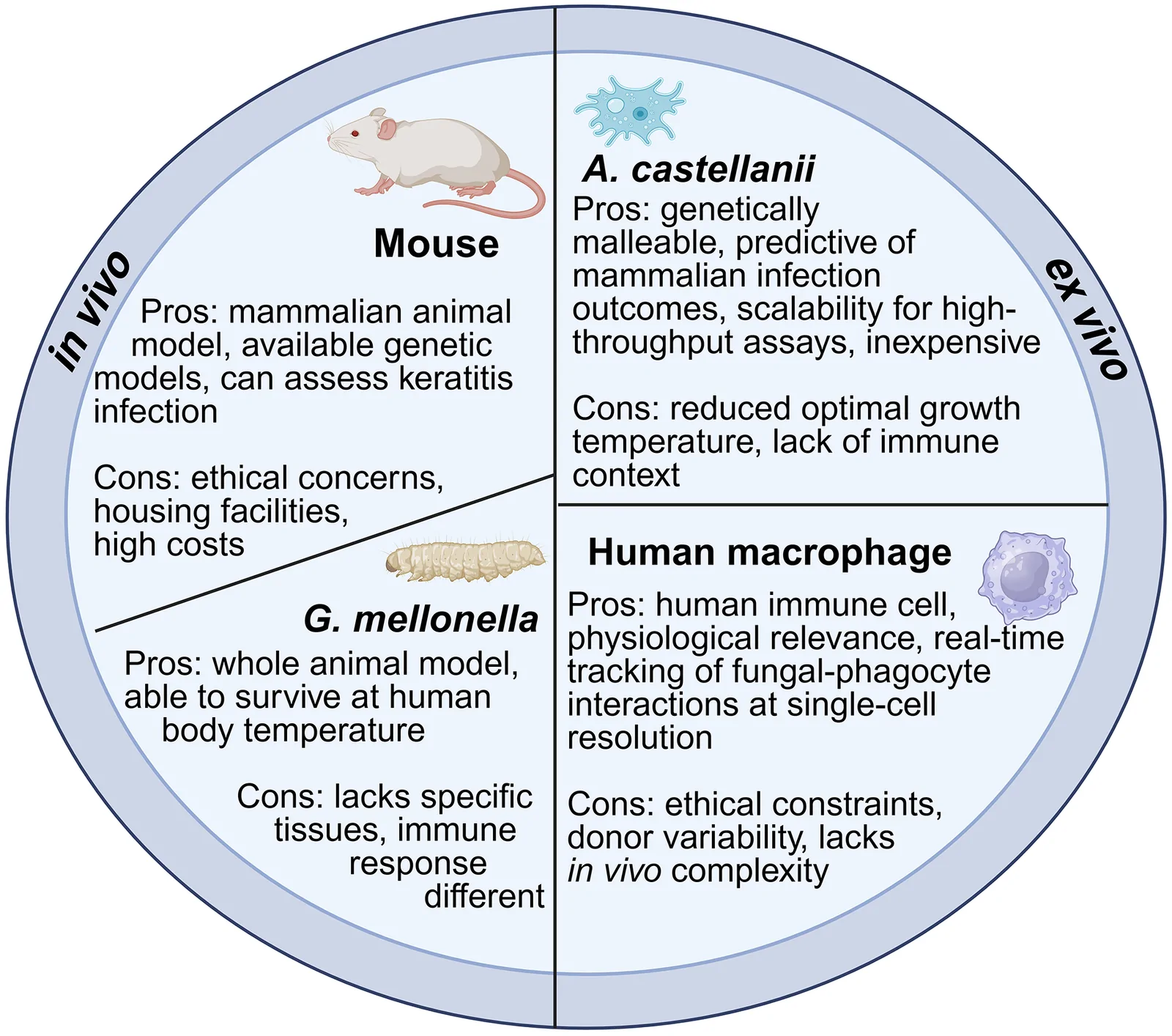
Summary of ex vivo and in vivo models to study invasive *Fusarium* infections. Figure created in BioRender.

To reduce animal usage, ex vivo models have also been applied to study fungal infectivity in mammalian hosts, with primary cells and macrophage cell lines representing some of the most widely used systems. For instance, primary human cornea fibroblasts [42] and epithelial cell lines [43] have been developed to assess fungal virulence factors associated with keratitis. Phagocytes constitute the first line of defense against invading fungal cells, and hence, the initial interaction between host phagocytes and fungal cells plays a crucial role in determining the outcome of infection [44,45]. Both primary macrophages and immortalized cell lines (e.g., THP-1, J774.1, or RAW 264.7) are commonly used to model these interactions [46]. Cell lines offer practical advantages, including ease of culture, reproducibility, and suitability for high-throughput screening. However, they often exhibit altered or attenuated immune responses compared to primary cells. In contrast, primary macrophages more faithfully recapitulate physiological immune signaling, including pattern recognition receptor engagement, cytokine production, and polarization states, but are limited by donor variability and experimental scalability. Among primary macrophages, both murine- and human-derived cells are widely used. Murine macrophages provide experimental accessibility and compatibility with in vivo models, whereas human macrophages offer greater translational relevance, capturing species-specific immune pathways that may influence host–pathogen interactions [47,48].

These systems have been instrumental in defining key aspects of *Fusarium*–phagocyte interactions. For example, live-cell video microscopy of J774.1 macrophages challenged with *F. oxysporum* germlings showed that macrophages migrate toward and engulf the fungus with kinetics similar to those previously reported only for *Candida albicans*, with comparable engulfment times (~6.7 min) and uptake efficiency (~95% to 96% of bound cells internalized within 15 min) [49]. Notably, macrophages migrated more rapidly toward *F. oxysporum* germlings than toward *C. albicans*. Following internalization, continued hyphal growth caused macrophage lysis and fungal escape. Furthermore, intracellular *F. oxysporum* arrested macrophage cytokinesis, producing multinucleated daughter cells that remained connected by the fungal hypha, a phenotype not described for *C. albicans*. Species-level comparisons using primary human polymorphonuclear leukocytes and monocyte-derived macrophages further showed that hyphal damage scales with effector-to-target ratio, but that *F. solani* hyphae resist immune-mediated killing more than both *F. oxysporum* and the weakly pathogenic mold *Verticillium nigrescens*, suggesting resistance to phagocyte-mediated killing correlates with the pathogenic potential of *Fusarium* species rather than representing a generic characteristic of filamentous fungi [50]. Together, these findings highlight the dynamic interplay between fungal morphogenesis, immune activation, and host cell damage that shapes the outcome of fusariosis at the cellular level, and begin to distinguish *Fusarium*-specific evasion strategies from those documented for other fungal pathogens.

Demonstrating a striking similarity in the infection process, environmental amoebas have also been used as powerful models to study the ecology and cellular microbial-phagocyte interactions. The free-living amoeba *Acanthamoeba castellanii*, which grows optimally at ~30 °C and remains viable at higher temperatures in some strains, has been widely exploited as an alternative system for studying fungal–phagocyte interactions at ambient conditions [51]. Amoebae and macrophages share conserved features such as phagocytosis, phagosome maturation, and microbicidal processes, enabling modeling of key host–pathogen interactions. In this context, *A. castellanii* provides a complementary platform that captures aspects of the environmental and ecological interactions that may shape microbial virulence prior to encounter with mammalian hosts.

Studies using this model have provided insights into *Fusarium* ecology and host interaction dynamics. *F. solani* conidia are readily internalized by *A. castellanii* but remain viable and can germinate intracellularly, with emerging hyphae eventually breaching the trophozoite, indicating that this species actively resists rather than merely tolerates amoebal predation [51]. Moreover, co-culture with live amoebae, as well as exposure to amoebal lysates or conditioned media, significantly enhances fungal growth, suggesting that amoeba-derived factors can promote *Fusarium* proliferation independently of direct cell contact. Interestingly, these interactions appear to be mutually beneficial in a strain-dependent manner. Exposure to *F. solani* increased encystment in some *A. castellanii* strains and enhanced chlorhexidine resistance in another, whereas passage of *F. solani* through amoeba increased its cytopathic activity toward mammalian cells, indicating a potentially mutualistic or permissive relationship rather than a purely predatory one. These findings extend the amoeboid predator–fungal virulence hypothesis, which proposes that environmental amoebae select for fungal traits promoting resistance to mammalian phagocytes [52]. In contrast to a purely predator–prey relationship, the *Fusarium*–*Acanthamoeba* interaction appears to confer reciprocal benefits to both organisms. These findings support the idea that amoeba–fungus interactions in environmental niches may influence fungal fitness and persistence, and could have downstream implications for infection, including in the context of keratitis.

Both macrophage- and amoeba-based systems are amenable to real-time monitoring of fungal–phagocyte interactions at single-cell resolution, enabling dynamic analysis of processes such as uptake, intracellular survival, and escape through live-cell imaging and flow cytometry. These models are also compatible with medium- to high-throughput approaches, although amoeba-based systems offer practical advantages in scalability and cost. However, both systems represent reductionist models that do not recapitulate the full complexity of in vivo infection, lacking multicellular immune interactions, tissue-specific microenvironments, and systemic immune responses. This limitation is particularly pronounced for amoebae, which operate outside of a mammalian immune context and therefore provide a more distant approximation of host–pathogen interactions.

Another heterologous model that has proven useful in assessing *Fusarium* virulence has been the greater wax moth, *Galleria mellonella* [53]. This insect model can be maintained close to human physiological temperature and possesses an innate immune system, including phagocytic hemocytes that are similar to mammalian phagocytes and are able to ingest the fungal conidium, and antimicrobial effector responses, enabling the study of systemic host–pathogen interactions in a whole-organism context.

Studies using *Galleria* have provided important insights into *Fusarium* pathogenesis and host interactions, while highlighting a feature that is unusual among medically important fungi: the same *F. oxysporum* isolate is able to cause disease in tomato plants, immunosuppressed mice, and *Galleria*, providing a rare trans-kingdom model for directly comparing virulence mechanisms across plant, invertebrate, and mammalian hosts. Infection with this isolate results in dose-dependent killing of larvae at both 30 °C and 37 °C following microconidia injection, and heat-killed conidia fail to induce mortality, demonstrating that virulence depends on active fungal growth rather than toxic or structural properties of the inoculum. Imaging with GFP-labeled strains revealed extensive hyphal proliferation within the hemocoel, interactions with hemocytes, and eventual dissemination throughout the larval body [54]. Importantly, mutants exhibiting reduced virulence in murine infection models display comparable attenuation in *Galleria*, supporting the predictive value of this model for identifying mammalian virulence determinants. In parallel, FSSC clinical isolates exhibit high lethality in larvae regardless of their clinical origin, with macroconidia displaying greater virulence than microconidia [53]. Killing kinetics in this model correlate with disease severity observed in murine infections, further reinforcing its relevance. Importantly, antifungal treatment with amphotericin B significantly prolongs survival in infected larvae, validating *Galleria* as a platform for antifungal efficacy testing.

Compared with ex vivo cell-based systems, *Galleria* provides greater biological complexity while remaining experimentally tractable and cost-effective, allowing simultaneous assessment of fungal dissemination, host survival, and antifungal efficacy at the whole-organism level. However, it lacks adaptive immunity and does not fully recapitulate mammalian immune signaling, positioning it as an intermediate model between reductionist cell-based systems and vertebrate infection models.

## Concluding remarks

The compartmentalized genome organization of clinical *Fusarium* isolates presents an investigation focal point to understand the contribution of accessory genes to human infection and to elucidate virulence mechanisms of this cross-kingdom pathogen. The completion of the genomes of several clinical isolates and the establishment of in vivo and ex vivo infection models offer tremendous opportunities to study this group of critically understudied medically important fungal pathogens. However, there are challenges and many unanswered questions that the *Fusarium* research community is facing. We know there are shared accessory genes among several related STs. Will more distantly related STs also share these potential virulence factors, or is the ability to infect mammalian hosts derived from convergent evolution? Even though the FOSC community is adapting the fusariosis strain NRRL 32931 [16], the keratitis strain MRL8996 [21], and the common ST33 strain FoCI-2 [20] as models to study infections [22], genotyping additional strains is necessary to understand the diversity of this highly dynamic pathogen group. One challenge, and also an opportunity, is the compartmentalized nature of FOSC genomes. Since all FOSC genomes share most of their genes as part of the core genome, it is desirable and possible to have a database that links annotations derived from individual genome projects to a unified nomenclature based on orthologs. Understanding the intrinsic antifungal resistance among this group of pathogens is another pressing question. With the growing population that is immunocompromised, there is an urgent need to develop effective anti-Fusaria drugs based on the understanding of their virulence and the mechanisms of intrinsic antifungal resistance.
